# Genomics and Prognosis Analysis of N^6^-Methyladenosine Regulators in Lung Adenocarcinoma

**DOI:** 10.3389/fgene.2021.746666

**Published:** 2021-12-09

**Authors:** Yanpin Ma, Huping Zhang

**Affiliations:** ^1^ Department of Oncology, The First Affiliated Hospital, and College of Clinical Medicine of Henan University of Science and Technology, Luoyang, China; ^2^ Department of Infectious Diseases, The First Affiliated Hospital, and College of Clinical Medicine of Henan University of Science and Technology, Luoyang, China

**Keywords:** lung adenocarcinoma, N6-methyladenosine, molecular subtypes, risk score, prognosis, tumor immunity

## Abstract

**Objective:** N^6^-methyladenosine (m^6^A) modification is involved in modulating various biological processes in human cancers. But the implication of m^6^A modification in lung adenocarcinoma (LUAD) is still unclear. Hence, this study conducted a comprehensive analysis of the expression and clinical implication of m^6^A regulators in LUAD.

**Methods:** Consensus clustering analysis of 502 LUAD samples in the TCGA dataset was presented based on the expression profiles of 20 m^6^A regulators using ConsensusClusterPlus package. Overall survival (OS), activation of signaling pathways and tumor immunity (immune/stromal score, tumor purity, expression of HLA and immune checkpoints, and immune cell infiltration) were compared between m^6^A modification patterns. The m^6^A-related genes between patterns were identified and prognostic m^6^A-related genes were imported into LASSO-cox regression analysis. The m^6^A risk score was developed and its prognostic implication was evaluated and externally verified in the GSE30219 and GSE72094 dataset. Furthermore, a nomogram that contained independent prognostic indicators was established, followed by external verification.

**Results:** Two m^6^A modification patterns were clustered across LUAD based on the expression similarity of the m^6^A regulators via consensus clustering analysis, with distinct OS, activation of signaling pathways and tumor immunity. Totally, 213 m^6^A-related genes that were identified by comparing two patterns were significantly related to LUAD prognosis. By LASSO method, we constructed the m^6^A risk score that was a reliable and independent prognostic factor for LUAD. Patients with low m^6^A risk score displayed a prominent survival advantage. After incorporating independent clinical features, we developed the prognostic nomogram that exhibited high predictive accuracy and the best clinical net benefit for OS.

**Conclusion:** Collectively, our study may provide a clinically useful tool for precise prognostic management and optimization of immunotherapeutic strategies for LUAD patients.

## Introduction

Lung cancer has the high incidence and mortality globally, occupying almost 20% of cancer-related deaths in 2018 (Bray et al., 2018). It was estimated that there were 2.1 million new lung cancer cases and 1.8 million deaths in 2018 (Bray et al., 2018). This disease mainly includes two histological subtypes: non-small cell lung cancer (NSCLC; 85%) and small cell lung cancer (SCLC). NSCLC contains lung adenocarcinoma (LUAD) and lung squamous cell carcinoma (Zhu et al., 2019). LUAD is the main histology, and its incidence is constantly on the rise. Conventional therapeutic options against NSCLC include surgery resection, chemotherapy, and radiotherapy. Despite the progress in combined and personalized therapies such as tyrosine kinase inhibitors and immunotherapies (PD1/PD-L1 inhibitors), the 5-year survival rate is only 16% (Zhang C. et al., 2020). Diagnosis of LUAD usually occurs at an advanced stage, and most patients experience badly toxic treatment and poor clinical benefit (Schmidt et al., 2019). Hence, it is of importance to explore specific prognostic models for predicting patients’ survival, which can assist design appropriate therapeutic strategies and management choice for distinct LUAD subgroups.

N^6^-methyladenosine (m^6^A) is the most abundant type of RNA post-transcriptional modification in eukaryotes, which plays a key role in a variety of biological processes by regulating the translation, splicing, stabilization, and degradation of mRNAs (Zhang H. et al., 2020). Typically, m^6^A regulators contain three types: writers (including VIRMA, METTL14, METTL3, RBM15, RBM15B, RBMX, WTAP, and ZC3H13), erasers (including ALKBH5 and FTO) and readers (including HNRNPA2B1, HNRNPC, IGF2BP1, IGF2BP2, IGF2BP3, YTHDC1, YTHDC2, YTHDF1, YTHDF2, and YTHDF3) (Fu et al., 2014). Emerging evidence suggests that the 20 m^6^A regulators display tight relationships with LUAD (Li F. et al., 2020; Chao et al., 2020; Li Y. et al., 2020). For instance, YTHDC2 suppresses LUAD carcinogenesis through inhibiting SLC7A11-dependent antioxidant function (Ma et al., 2021). Moreover, FTO accelerates LUAD progression through mRNA demethylation (Ding et al., 2020). ALKBH5 facilitates proliferation and invasion of LUAD cells following intermittent hypoxia (Chao et al., 2020). YTHDF1 is linked to hypoxia adaptation and LUAD progression (Shi et al., 2019). FTO triggers LUAD progression through activating cell migration via mRNA demethylation (Ding et al., 2020). These experimental evidences suggest that an in-depth understanding of m^6^A regulators may deepen our understanding on the role of m^6^A modification in the progression of LUAD. Here, we comprehensively analyzed the expression and clinical implication of m^6^A regulators in LUAD.

## Materials and Methods

### Dataset Preparation

The Cancer Genome Atlas database (TCGA) RNA-seq data (FPKM values) and matched clinical features of 502 LUAD patients were retrieved from the Genomic Data Commons website (https://portal.gdc.cancer.gov/). The FPKM values were normalized with transcripts per million (TPM) method, followed by log2 conversion. Microarray expression profiling and clinical information of 274 LUAD samples and 398 LUAD samples were separately obtained from the GSE30219 dataset (Rousseaux et al., 2013) and the GSE72094 dataset (Schabath et al., 2016) in the Gene Expression Omnibus (GEO) database (https://www.ncbi.nlm.nih.gov/geo/). All data were obtained from the publicly available databases. Therefore, it was not applicable for the ethical approval. A total of 20 m^6^A regulators including 8 writers (VIRMA, METTL14, METTL3, RBM15, RBM15B, RBMX, WTAP, and ZC3H13), 2 erasers (ALKBH5 and FTO), and 10 readers (HNRNPA2B1, HNRNPC, IGF2BP1, IGF2BP2, IGF2BP3, YTHDC1, YTHDC2, YTHDF1, YTHDF2, and YTHDF3) were collected from the published literature. The location of these m^6^A regulators on the human chromosomes was plotted through Rcircos package (version 1.2.1) (Zhang et al., 2013). Protein-protein interaction analysis of the m^6^A regulators was performed by the STRING online database (version: 11.0; https://string-db.org/) (Szklarczyk et al., 2017).

### Consensus Clustering Analysis

Consensus clustering analysis was carried out utilizing ConsensusClusterPlus package (version 1.48.0) to assign LUAD patients in the TCGA dataset into different m^6^A modification patterns with 50 iterations and resample rate of 80% based on the expression matrix of the 20 m^6^A regulators (Wilkerson and Hayes, 2010). Kaplan-Meier curves of overall survival (OS) were conducted between two m^6^A modification patterns. The survival difference was compared with log-rank test. The t-distributed stochastic neighbor embedding (t-SNE) was presented to validate the accuracy of this classification.

### Gene Set Variation Analysis

The activation of pathways was quantified in each LUAD sample from the TCGA dataset by single-sample gene set enrichment analysis (ssGSEA) method derived from GSVA package (version 1.32.0) in an unsupervised manner (Hänzelmann et al., 2013). The gene set of “c2. cp.kegg.v7.2. symbols” was obtained from the Molecular Signatures Database, which was used as the reference set (Liberzon et al., 2015).

### Estimation of Tumor Immunity

According to the normalized expression matrix, stromal and immune scores across LUAD samples in the TCGA dataset were estimated via the Estimation of Stromal and Immune Cells in Malignant Tumors Using Expression Data (ESTIMATE) method (https://sourceforge.net/projects/estimateproject/). (Yoshihara et al., 2013) that was applied for inferring the overall infiltrations of stromal and immune cells in LUAD tissues based on gene symbols. The tumor purity was calculated via ESTIMATE and consensus measurement of purity estimations methods. Tumor immune signatures were assessed in LUAD samples, including the mRNA expression of human leukocyte antigen (HLA) family genes and immune checkpoints. The infiltration levels of immune cells were quantified across LUAD samples based on the published gene signatures utilizing the ssGSEA algorithm (Charoentong et al., 2017; Jia et al., 2018).

### Identification of m^6^A-Related Differentially Expressed Genes

The DEGs were screened between two m^6^A modification patterns in the TCGA dataset through limma package (version 3.40.6) (Ritchie et al., 2015). The cut-off was |log2 fold change (FC)|>1 and false discovery rate (FDR) < 0.001. FDR was calculated with Benjamin–Hochberg method. The m^6^A-related DEGs were visualized into volcano and heat maps via pheatmap package (version 1.0.12).

### Construction of a Least Absolute Shrinkage and Selection Operator-Cox Regression Model

LASSO represents a regularization and descending dimension method that has been applied for prognostic Cox models. Univariate-cox regression analysis was utilized to assess the correlation between overall survival (OS) of LUAD patients in the TCGA dataset and the m^6^A-related DEGs. The genes with *p* < 0.05 were input into the LASSO-cox regression model through glmnet package (version 2.0.16) (Engebretsen and Bohlin, 2019). Variable selection was presented for penalizing the data fitting criteria, which reduced the complexity and made the model more interpretable. The coefficient of each variable was the average estimate of the coefficient obtained from 10-fold cross-verification. The m^6^A risk score was developed following the formula: $\text{risk}\;\text{score}=\sum_{i=1}^{n}Coef\;\left(i\right)X\;\left(i\right)$ , where n indicated the number of variables in this LASSO model, Coef 1) represented the regression coefficient, and X 1) meant the mRNA expression levels of variables in LUAD samples. To evaluate the prediction utility of the LASSO model, time-dependent receiver operating characteristic (ROC) curves were conducted by survivalROC package (version 1.0.3) in the TCGA, GSE30219 and GSE72094 datasets, followed by calculation of one, three and 5-year area under curve (AUC). In the two datasets, LUAD patients were separately split into two groups according to the median m^6^A risk score through survminer (version 0.4.9) and survival (version 3.2–13) packages. Kaplan-Meier curves of OS were depicted for two groups via survival package and OS difference was compared with log-rank test. The distribution of survival status in two groups was then visualized. By pheatmap package, heatmap was established to visualize the mRNA expression pattern of each variable in the LASSO model.

### Estimation of the Prediction Independency of the m^6^A Risk Score

To estimate whether the m^6^A risk score independently predicted LUAD patients’ OS, univariate- and multivariate-cox regression analysis was carried out following adjusting clinical features (gender, stage, T, N and M) in the TCGA, GSE30219 and GSE72094 datasets. Hazard ratio (HR) and *p* value were calculated for each variable.

### Construction of a Nomogram Model

To better apply the m^6^A risk score in clinical practice, a nomogram that included independent prognostic indicators was conducted to predict LUAD patients’ one, three and 5-year OS in the TCGA, GSE30219 and GSE72094 datasets via rms package (version 6.2–0). Calibration plot was presented to evaluate predictive performance of the m^6^A risk score. Furthermore, decision curve analysis was carried out for calculating the clinical net benefit of every model in comparison to all or none strategies. The none plots indicated the assumption that no subjects had one, three or 5-year OS. Meanwhile, all plots indicated the assumption that each subject had one, three or 5-year OS at specific threshold probabilities. The best model was the one with the highest net benefit.

### Statistical Analysis

All statistical analysis was implemented through the R software (version 3.6.3). Wilcoxon test was used for comparison between two groups. *p* < 0.05 was statistically significant.

## Results

### Landscape of Expression and Prognostic Implications of m^6^A Regulators in Lung Adenocarcinoma

**FIGURE 1:**
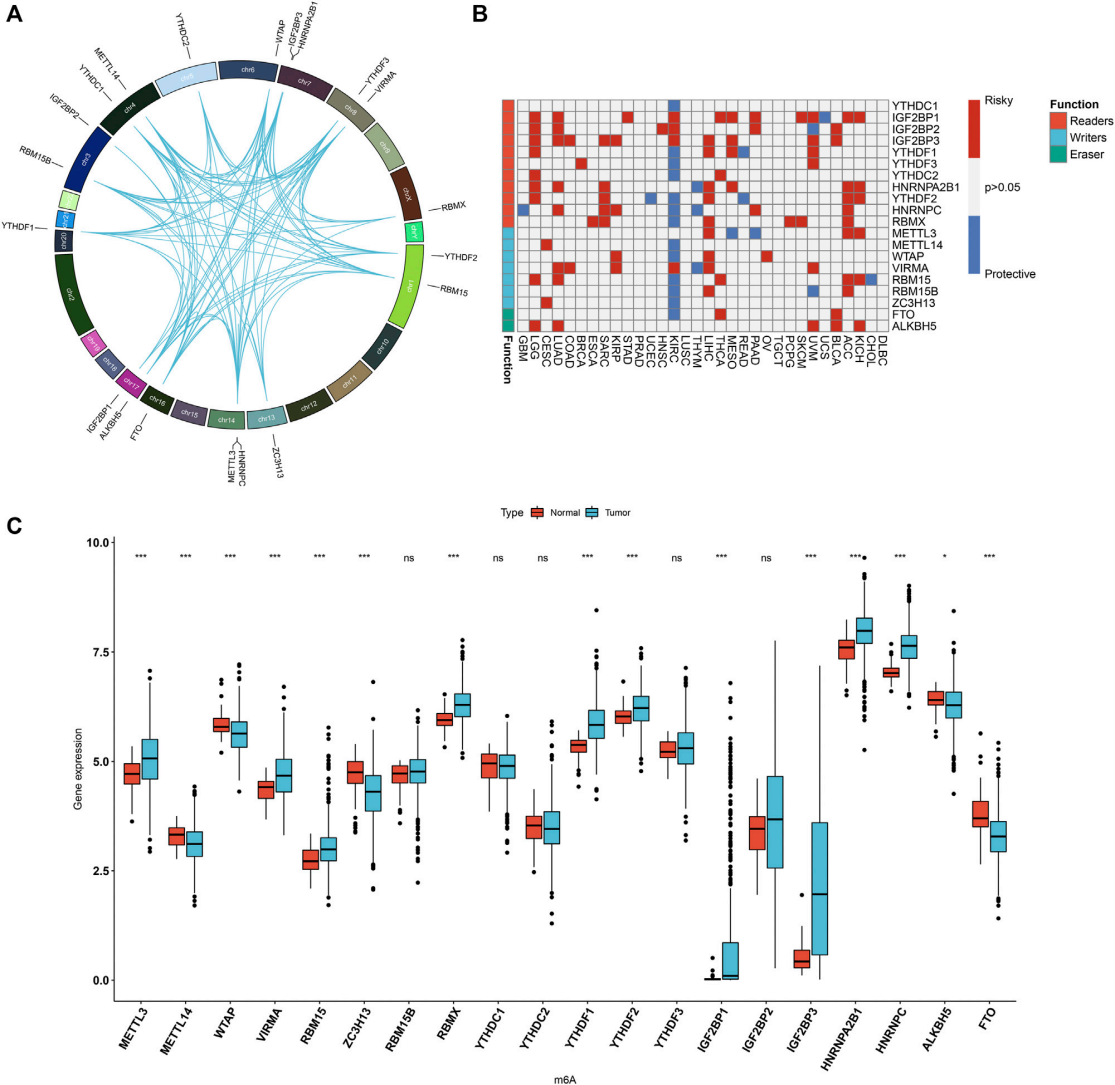
Landscape of expression patterns and prognostic value of 20 m^6^A regulators in LUAD. **(A)** The location of 20 m^6^A regulators on the human chromosomes. Lines in circle represented their interactions according to the STRING database. **(B)** Heatmap showing the correlations between m^6^A regulators and prognosis of pan-cancer in TCGA cohorts. The left side of the heatmap showed the functions (writer, eraser and reader) of m^6^A regulators. Red indicated that a specific regulator was a risk factor for a type of cancer and blue indicated that a specific regulator was a protective factor for a type of cancer. **(C)** Comparison of the expression of m^6^A regulators between LUAD and normal tissues in the TCGA dataset with Wilcoxon test. Ns: not significant; *p < 0.05; ***p < 0.001.

Totally, 20 m^6^A regulators including 8 writers (VIRMA, METTL14, METTL3, RBM15, RBM15B, RBMX, WTAP, and ZC3H13), 2 erasers (ALKBH5 and FTO), and 13 readers (HNRNPA2B1, HNRNPC, IGF2BP1, IGF2BP2, IGF2BP3, YTHDC1, YTHDC2, YTHDF1, YTHDF2, and YTHDF3) were collected in this study. Figure 1A and Supplementary Table S1 depicted their location on the chromosomes. Also, there were close direct (physical) or indirect (functional) interactions between the m^6^A regulators (Figure 1A). Pan-cancer analysis revealed the prognostic implication of the m^6^A regulators in the TCGA cohort (Figure 1B). For LUAD, IGF2BP1, IGF2BP2, IGF2BP3, HNRNPA2B1, HNRNPC, VIRMA, RBM15 and ALKBH5 were significant risk factors. The mRNA expression of the 20 m^6^A regulators was compared between LUAD and normal tissues. We found that METTL3, VIRMA, RBM15, RBMX, YTHDF1, YTHDF2, IGF2BP1, IGF2BP3, HNRNPA2B1 and HNRNPC displayed higher mRNA expression in LUAD compared to normal specimens (Figure 1C). Meanwhile, METTL14, WTAP, ZC3H13, ALKBH5 and FTO were significantly down-regulated in LUAD than normal tissues. These data were indicative of the important implication of m^6^A regulators in the progression of LUAD.

### Construction of m^6^A Regulators-Mediated m^6^A Modification Patterns in Lung Adenocarcinoma

**FIGURE 2:**
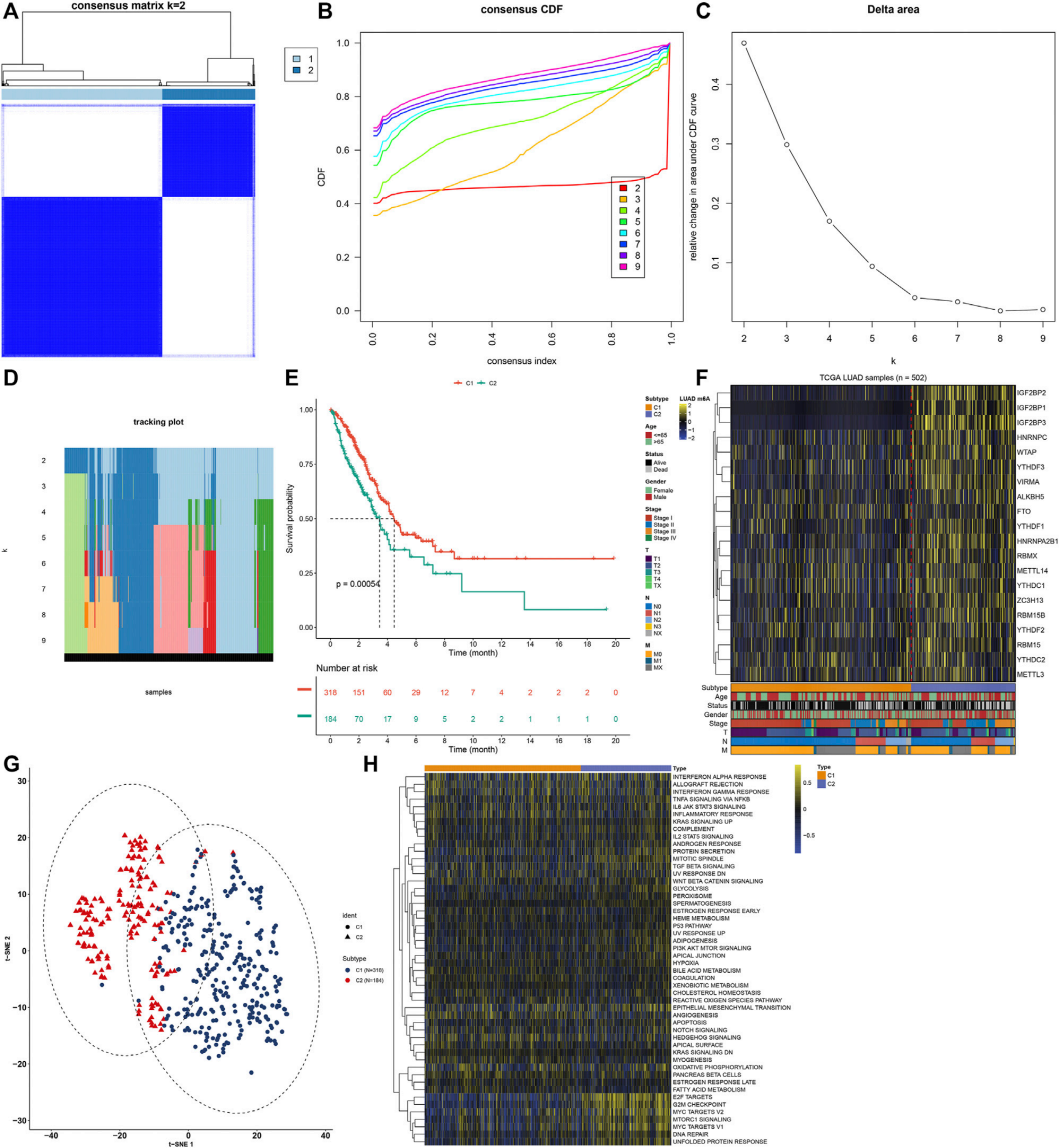
Construction of m^6^A regulator-mediated m^6^A modification patterns in LUAD from the TCGA cohort. **(A)** Consensus matrix when number of groups (k) = 2. In the consensus matrix, white meant that samples were impossibly clustered together, and dark blue meant that samples were always clustered together. Both rows and columns of the matrix represented samples. **(B)** Consensus cumulative distribution function (CDF) diagram when different k values. **(C)** Delta area plot for relative change in the area under CDF curve for k compared to k-1. **(D)** Tracking plot for sample clustering when different k values. **(E)** Kaplan-Meier curves of OS between two m^6^A modification patterns. The survival probabilities were compared with log-rank test. **(F)** Heatmap for the expression patterns of 20 m^6^A regulators in different m^6^A modification patterns, age, survival status, gender, stage, T, N and M. **(G)** The t-SNE plots for verifying the differences between two m^6^A modification patterns. **(H)** GSVA for the activation of signaling pathways in LUAD samples between two m^6^A modification patterns.

A total of 502 LUAD samples were clustered based on the expression similarity of the m^6^A regulators via consensus clustering analysis. Our data found that when the number of groups (k) = 2, there was an excellent clustering among LUAD samples in the consensus matrix (Figure 2A). Consensus cumulative distribution function (CDF) diagram showed that when k value = 2, CDF reached an approximate maximum (Figure 2B). Delta area plot depicted the relative change in the area under CDF curve for k compared to k-1 (Figure 2C). As shown in tracking plot, when k value = 2, sample classification was stable (Figure 2D). Hence, we clustered LUAD patients into two m^6^A modification patterns, named as C1 (N = 318) and C2 (N = 184). To further understand the characteristics of m^6^A modification patterns clustered by consensus clustering analysis in LUAD, we firstly analyzed the difference in OS. The data showed that C2 exhibited a more unfavorable OS in comparison to C1 (*p* = 0.00054; Figure 2E). Furthermore, we visualized the expression patterns of the m^6^A regulators in two m^6^A modification patterns. As shown in Figure 2F, IGF2BP2, IGF2BP1, IGF2BP3 had distinctly higher expression in C2 compared to C1. The t-SNE was carried out for verifying whether the categories were appropriate. Our results showed that most of samples from C1 and C2 were separately gathered (Figure 2G), indicating that the clustering of two m^6^A modification patterns was a relatively good choice. By applying GSVA algorithm, activation of several signaling pathways was quantified in each LUAD sample. We found that E2F targets, G2M checkpoint, MYC targets, mTORC1 signaling, DNA repair and unfolded protein response had higher activations in C2 than C1 (Figure 2H).

### Two m^6^A Modification Patterns Characterized by Distinct Tumor Immunity

**FIGURE 3:**
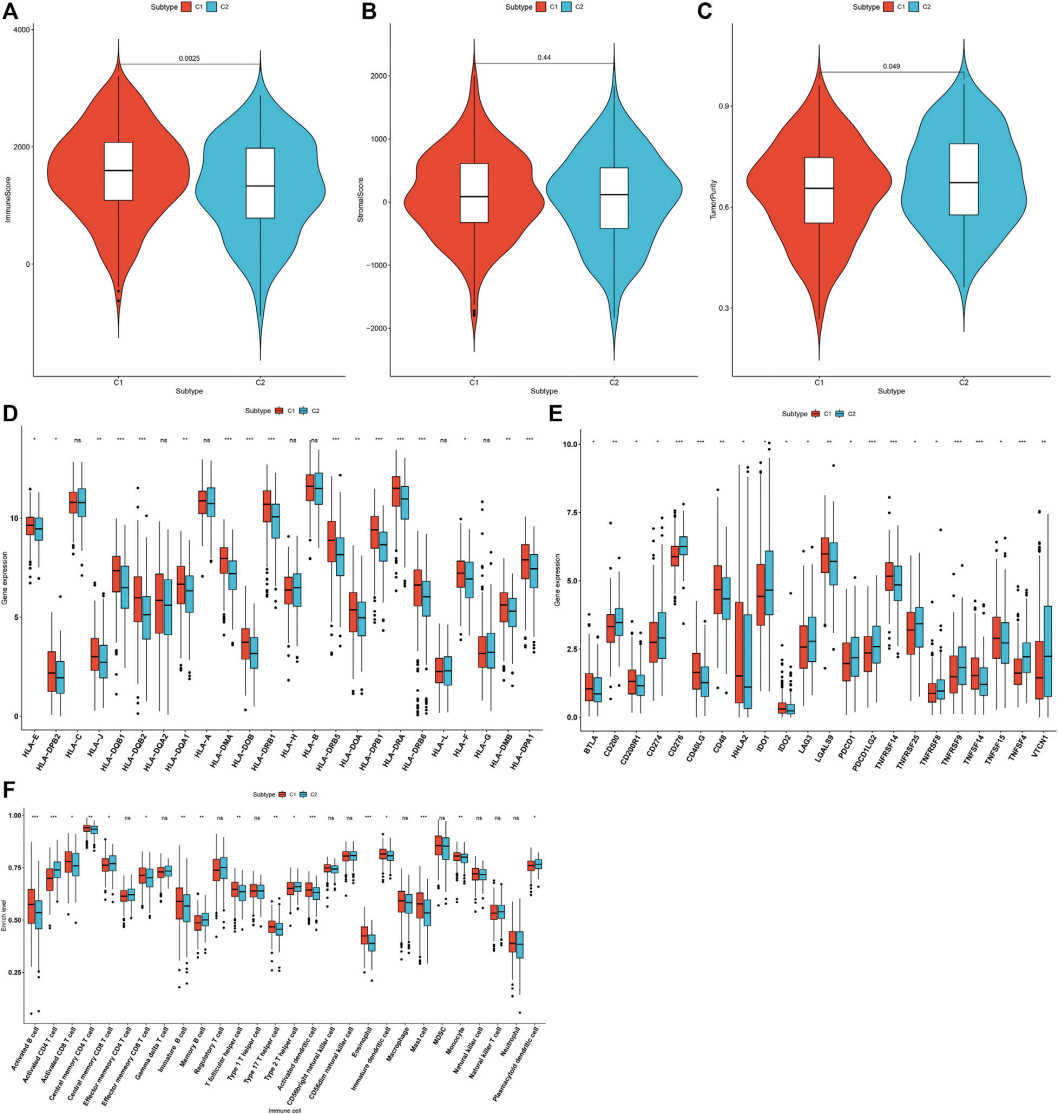
Two m^6^A modification patterns characterized by distinct tumor immunity across 502 LUAD specimens from the TCGA cohort. **(A–C)** Quantification of immune score, stromal score, and tumor purity in LUAD samples from two m^6^A modification patterns through ESTIMATE algorithm. **(D)** Comparison of the mRNA expression of HLA genes between two m^6^A modification patterns. **(E)** Comparison of the mRNA expression of immune checkpoints between two m^6^A modification patterns. **(F)** Quantification of the infiltration levels of immune cells in LUAD samples from two m^6^A modification patterns via ssGSEA algorithm. Ns: not significant; *p < 0.05; **p < 0.01; ***p < 0.001.

The overall infiltration levels of immune and stromal cells were estimated in 502 LUAD samples from the TCGA dataset via ESTIMATE algorithm. Compared to C1, C2 pattern had a significantly decreased immune score (*p* = 0.0025; Figure 3A). But no significant difference in stromal score was detected between m^6^A modification patterns (Figure 3B). There was significantly increased tumor purity in C2 than C1 (*p* = 0.049; Figure 3C). The mRNA expression of HLA genes and immune checkpoints was compared between m^6^A modification patterns. Most of HLAs were highly expressed in C1 compared to C2, including HLA-E, HLA-DPB2, HLA-J, HLA-DQB1, HLA-DQB2, HLA-DQA1, HLA-DMA, HLA-DOB, HLA-DRB1, HLA-DRB5, HLA-DOA, HLA-DPB1, HLA-DRA, HLA-DRB6, HLA-F, HLA-DMB and HLA-DPA1 (Figure 3D). We also evaluated the differences in mRNA expression of common immune checkpoints between two m^6^A modification patterns. Our results showed that BTLA, CD200R1, CD40LG, CD48, HHLA2, IDO2, LGALS9, TNFRSF14, TNFSF14 and TNFSF15 displayed higher mRNA expression in C1 compared to C2 (Figure 3E). Meanwhile, C2 pattern had increased mRNA expression of CD200, CD274, CD276, IDO1, LAG3, PDCD1, PDCD1LG2, TNFRSF25, TNFRSF8, TNFRSF9, TNFSF4 and VTCN1 in comparison to C1. The infiltration levels of immune cells were quantified in each LUAD specimen via ssGSEA algorithm. Compared to C2, there were increased infiltration levels of activated B cell, activated CD8 T cell, central memory CD4 T cell, effector memory CD8 T cell, immature B cell, T follicular helper cell, type 17 T helper cell, activated dendritic cell, eosinophil, immature dendritic cell, mast cell and monocyte in C1 (Figure 3F). The higher infiltration levels of activated CD4 T cell, central memory CD8 T cell, memory B cell, type 2 T helper cell and plasmacytoid dendritic cell were found in C2 than C1.

### Identification of DEGs Between Two m^6^A Modification Patterns

**FIGURE 4:**
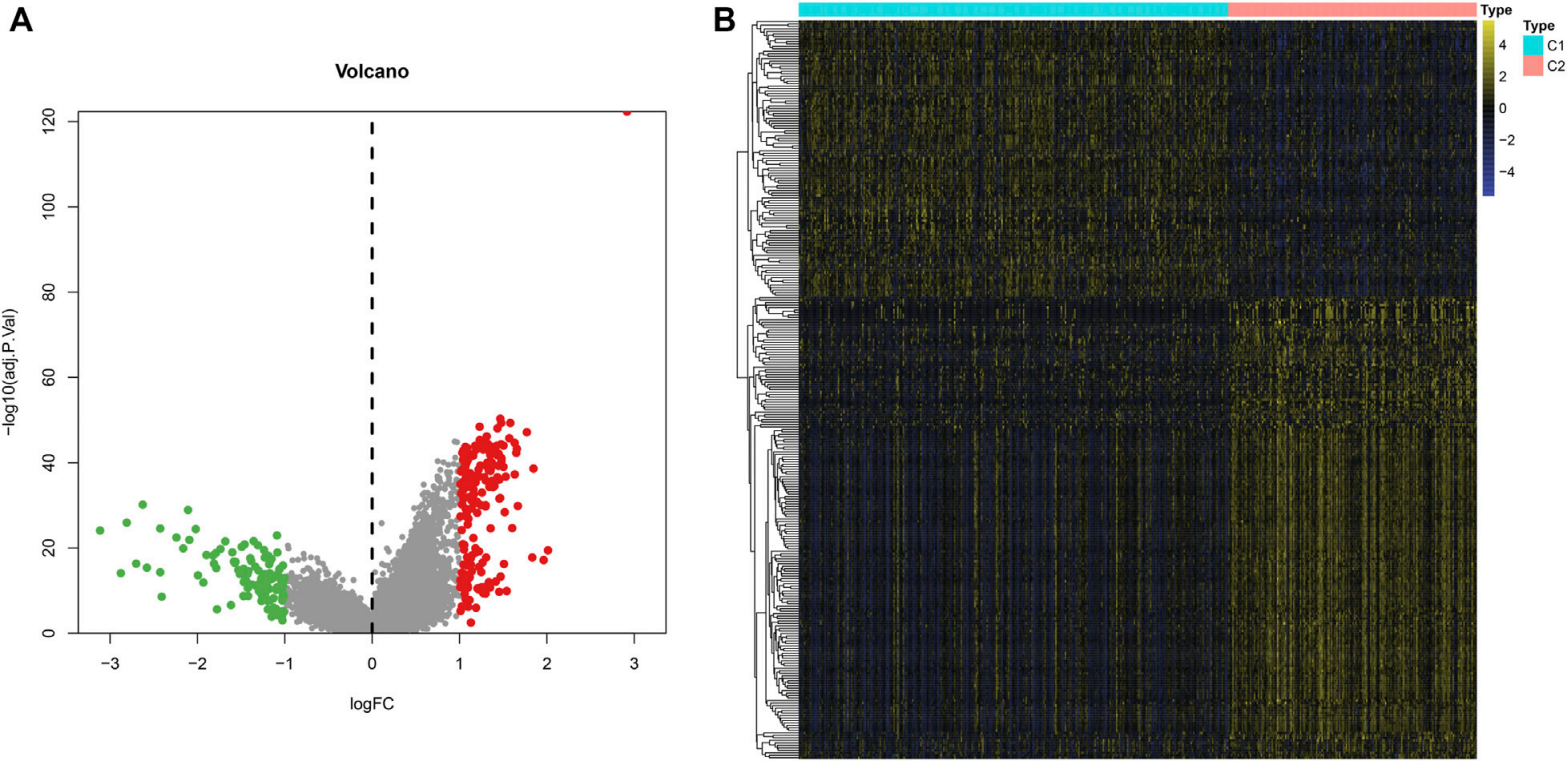
Identification of DEGs between two m^6^A modification patterns across LUAD samples from the TCGA dataset. **(A)** The volcano map of 297 genes that were abnormally expressed between two m^6^A modification patterns. Red dots indicated genes with high expression in C1 and green dots represented genes with low expression in C1. **(B)** Hierarchical clustering analysis of the 297 m^6^A-related DEGs in LUAD samples from two m^6^A modification patterns.

To explore the molecular mechanisms underlying two m^6^A modification patterns, we presented differential expression analysis. With the cutoff of |log2FC|>1 and adjusted *p* < 0.001, a total of 297 genes exhibited abnormal expression between two m^6^A modification patterns (Figures 4A,B). Among them, 111 genes were down-regulated and 186 were up-regulated in C1 compared to C2 (Supplementary Table S2). These genes could be affected by m^6^A methylation modification in LUAD.

### Development of an m^6^A Risk Score for Lung Adenocarcinoma

**FIGURE 5:**
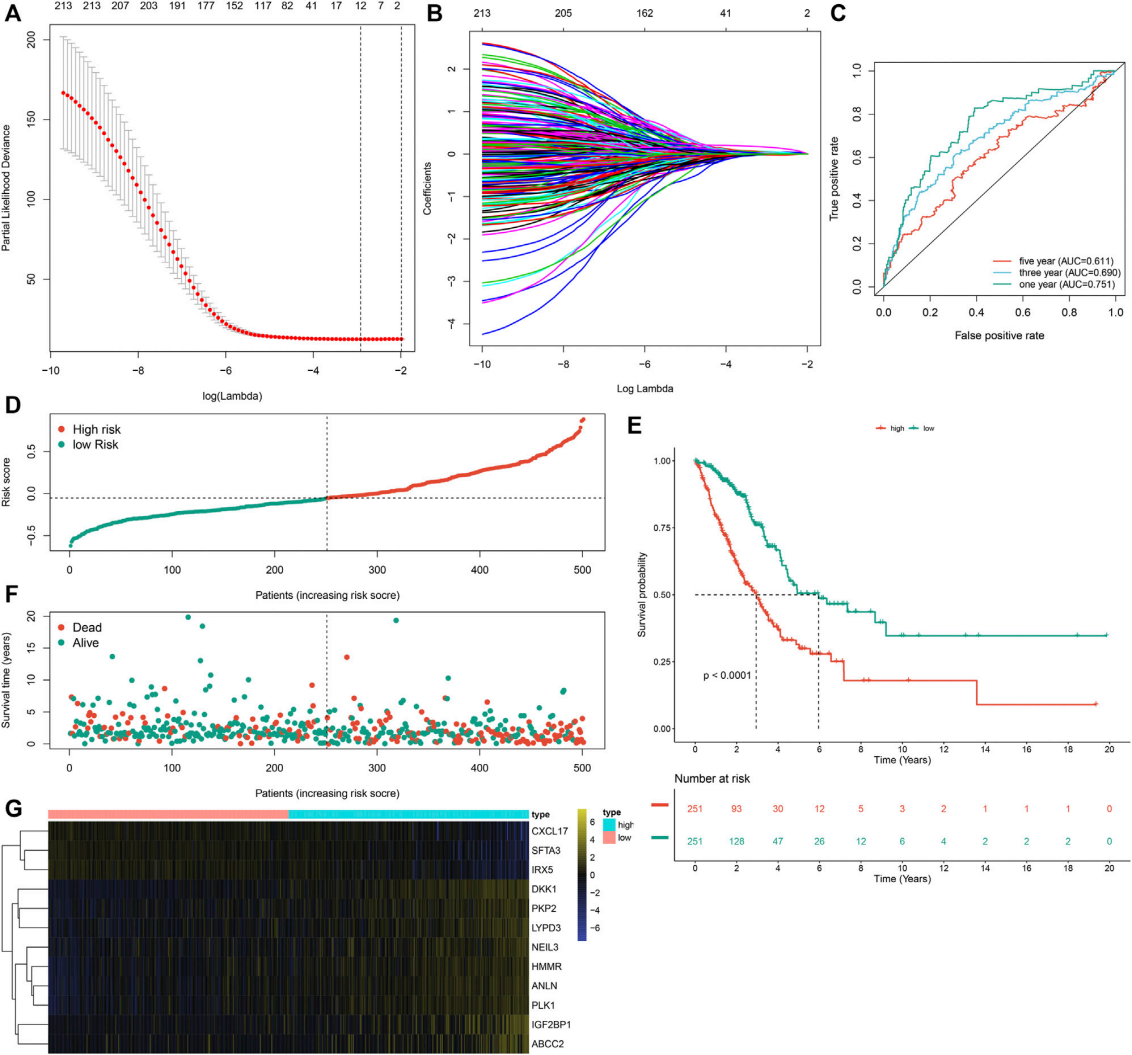
Development of an m^6^A risk score for LUAD by LASSO-cox regression analysis in LUAD samples from the TCGA cohort. **(A)** Partial likelihood deviance in each lambda value for the LASSO-cox regression analysis. **(B)** Process of variable selection in the LASSO-cox regression model by 10-fold cross-verification. **(C)** ROC curves under one, three and 5-year OS based on the m^6^A risk score. **(D)** The distribution of the m^6^A risk score and identification of the median m^6^A risk score indicated by vertical dotted line. **(E)** Kapan-Meier curves of OS for high- and low-m^6^A risk score groups, followed by log-rank test. **(F)** The distribution of survival status in high- and low-m^6^A risk score groups. Red dots indicated dead status and blue dots indicated alive status. **(G)** Hierarchical clustering analysis for the mRNA expression patterns in LUAD samples from high- and low-m^6^A risk score groups.

Prognostic implications of the 297 m^6^A-related DEGs were assessed via univariate-cox regression analysis. As a result, 213 genes had significant correlations to LUAD prognosis in the TCGA dataset (Supplementary Table S3). Candidate prognostic m^6^A-related DEGs were further screened with LASSO-Cox regression analysis (Figures 5A,B). As a result, 12 candidate m^6^A-related DEGs were identified for constructing a LASSO-cox regression model. Non-zero coefficients and the expression of 12 m^6^A-related DEGs in this LASSO-cox regression model were calculated in the TCGA dataset. The m^6^A risk score formula was as follows: m^6^A risk score = 0.0301860610758127 * mRNA expression of ANLN + 0.0263443393604996 * mRNA expression of PLK1 + 0.0576834829109261 * mRNA expression of IGF2BP1 + 0.0236368688862302 * mRNA expression of HMMR + 0.0356239951044486 * mRNA expression of NEIL3 + (-0.00157287764972551) * mRNA expression of SFTA3 + (-0.0250630515726943) * mRNA expression of CXCL17 + (-0.0244060686771379) * mRNA expression of IRX5 + 0.0277060677471147 * mRNA expression of PKP2 + 0.0281636442957098 * mRNA expression of LYPD3 + 0.00917559407956536 * mRNA expression of ABCC2 + 0.157371504648263 * mRNA expression of DKK1. ROC curves were conducted to evaluate whether the m^6^A risk score accurately and sensitively estimated the survival likelihood of LUAD patients in the TCGA dataset. The AUC values of one, three and 5-year OS were separately 0.751, 0.690 and 0.611 (Figure 5C). These indicated the good predictive performance of the m^6^A risk score. Figure 5D depicted the distribution of the m^6^A risk score across LUAD patients. According to the median m^6^A risk score, patients were split into high- and low-m^6^A risk score groups. The OS difference was compared between groups. As shown in Figure 5E, low m^6^A risk score group displayed a potential survival advantage in comparison to high m^6^A risk score group (*p* < 0.0001). The distribution of survival status was visualized in Figure 5F. We found that high m^6^A risk score group had the relatively increased number of dead status than low m^6^A risk score group. Figure 5G showed the mRNA expression of 12 variables in the model between high- and low-m^6^A risk score groups. DKK1, PKP2, LYPD3, NEIL3, HMMR, ANLN, PLK1, IGF2BP1 and ABCC2 displayed higher mRNA expression in high-m^6^A risk score group compared to low-m^6^A risk score group.

### External Verification of the m^6^A Risk Score in Lung Adenocarcinoma Prognosis

**FIGURE 6:**
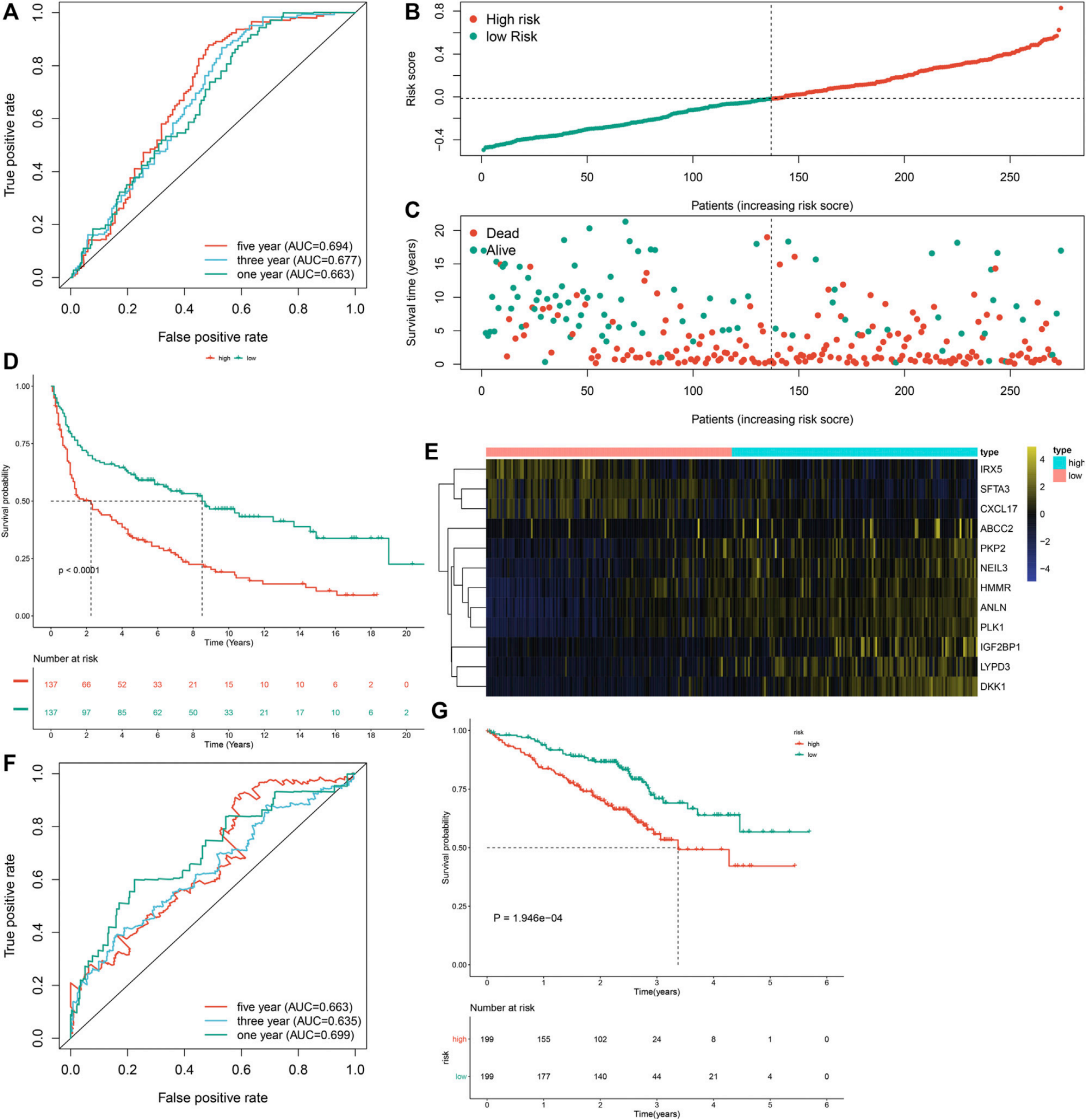
External verification of the m^6^A risk score for LUAD samples in the GSE30219 and GSE72094 datasets. **(A)** ROC curves for estimating the efficacy of the m^6^A risk score in prediction of one, three and 5-year OS in the GSE30219 dataset. **(B)** The distribution of m^6^A risk score and identification of the median m^6^A risk score indicated by vertical dotted line in the GSE30219 dataset. **(C)** The distribution of survival status in high- and low-m^6^A risk score groups. Red dots indicated dead status and blue dots indicated alive status in the GSE30219 dataset. **(D)** Kapan-Meier curves of OS for high- and low-m^6^A risk score groups, followed by log-rank test in the GSE30219 dataset. **(E)** Hierarchical clustering analysis for the mRNA expression patterns in LUAD samples from high- and low-m^6^A risk score groups in the GSE30219 dataset. **(F)** ROC curves for assessing the efficacy of the m^6^A risk score in prediction of one, three and 5-year OS in the GSE72094 dataset. **(G)** Kapan-Meier curves of OS for high- and low-m^6^A risk score groups, followed by log-rank test in the GSE72094 dataset.

To externally verify the prognostic implication of the m^6^A risk score, we acquired the transcriptome data and follow-up information of 274 LUAD patients from the GSE30219 cohort. The AUC values under one, three and 5-year OS were separately 0.663, 0.677 and 0.694 (Figure 6A). According to the median m^6^A risk score, we clustered these LUAD patients into two groups (Figure 6B). High m^6^A risk score group had more patients with dead status than low m^6^A risk score group (Figure 6C). Consistently, high m^6^A risk score was markedly associated with worse prognosis of LUAD in comparison to low m^6^A risk score (*p* < 0.0001; Figure 6D). Furthermore, DKK1, PKP2, LYPD3, NEIL3, HMMR, ANLN, PLK1, IGF2BP1, and ABCC2 were highly expressed in high m^6^A risk score group than low m^6^A risk score group **(** Figure 6E). We also verified the prognostic significance of the m^6^A risk score in the GSE72094 dataset. The AUC values at one, three and 5-year OS were 0.699, 0.635, and 0.663 (Figure 6F). As expected, high m^6^A risk score indicated poorer OS than low m^6^A risk score (Figure 6G). Therefore, the m^6^A risk score possessed the potential in accurately predicting survival outcomes of LUAD patients.

### The m^6^A Risk Score Acts as an Independent Prognostic Indicator of Lung Adenocarcinoma

**FIGURE 7:**
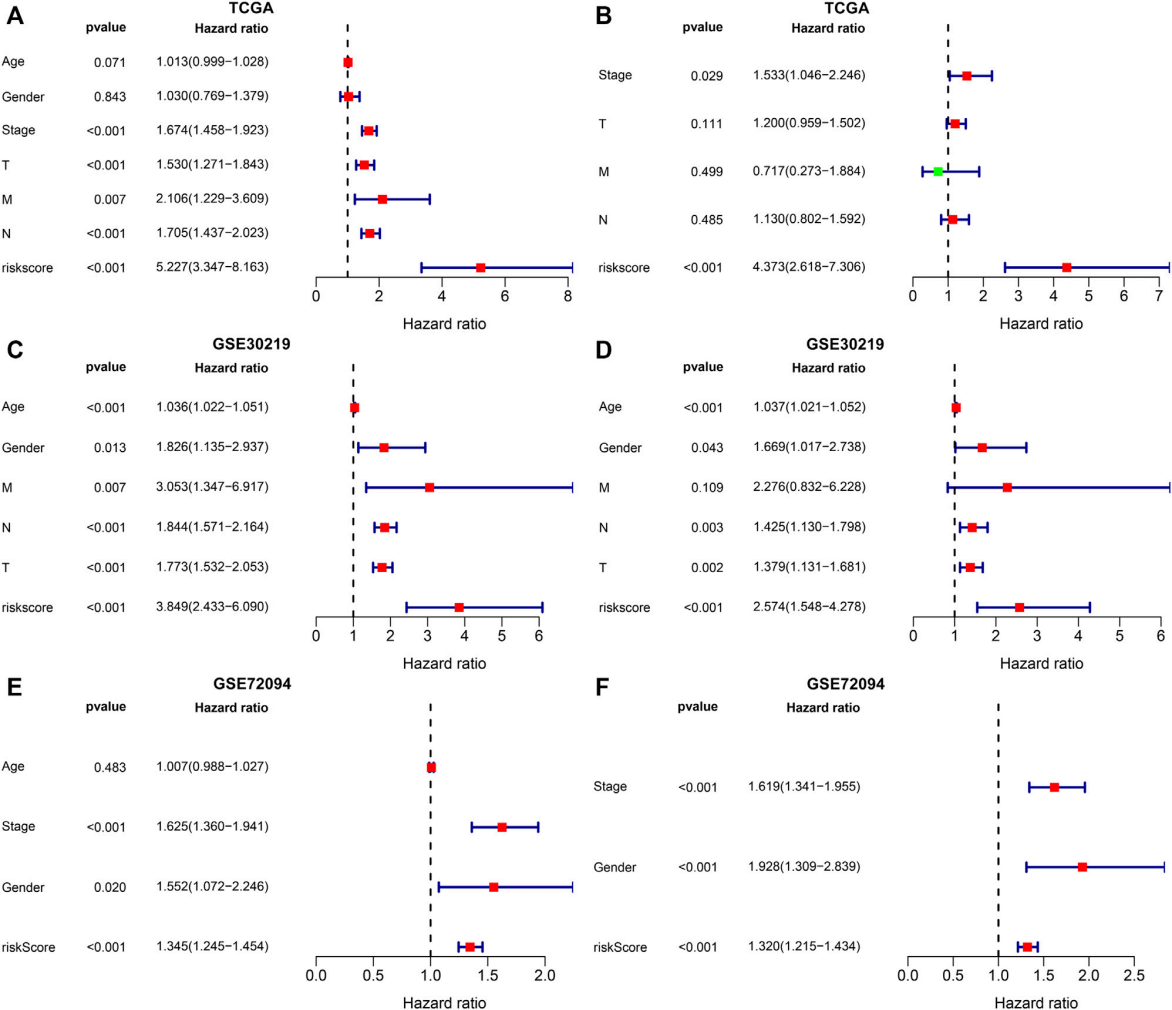
Evaluation and external verification of the predictive independency of the m^6^A risk score in LUAD patients. **(A)** Univariate-cox regression analysis for the correlations between the m^6^A risk score and LUAD patients’ OS in the TCGA dataset. **(B)** Multivariate-cox regression analysis for assessing the independent prognostic factors in the TCGA dataset. **(C)** External validation of the correlations between the m^6^A risk score and LUAD patients’ OS in the GSE30219 dataset by univariate-cox regression analysis. **(D)** External validation of the independent prognostic factors in the GSE30219 dataset through multivariate-cox regression analysis. **(E)** External verification of the relationships of the m^6^A risk score with LUAD patients’ OS in the GSE72094 dataset through univariate-cox regression analysis. **(F)** External verification of the independent prognostic factors in the GSE72094 dataset through multivariate-cox regression analysis.

In the TCGA dataset, univariate-cox regression analysis showed that the m^6^A risk score (*p* < 0.001; HR: 5.227 (3.347–8.163)), stage (*p* < 0.001; HR: 1.674 (1.458–1.923)), T (*p* < 0.001; HR: 1.530 (1.271–1.843)), N (*p* < 0.001; HR: 1.705 (1.437–2.023)) and M (*p* = 0.007; HR: 2.106 (1.229–3.609)) were significantly associated with LUAD prognosis (Figure 7A). These prognostic factors were input into multivariate-cox regression analysis. In Figure 7B, m^6^A risk score [*p* < 0.001; HR: 4.373 (2.618–7.306)] and stage [*p* = 0.029; HR: 1.533 (1.046–2.246)] were independent prognostic factors of LUAD. The prognostic implication of the m^6^A risk score was externally verified in the GSE30219 and GSE72094 cohorts. Our results confirmed that the m^6^A risk score could independently predict LUAD prognosis both in the GSE30219 cohort (Figures 7C,D) and GSE72094 cohort (Figures 7E,F).

### Establishment and Verification of a Prognostic Nomogram for Lung Adenocarcinoma Patients

**FIGURE 8:**
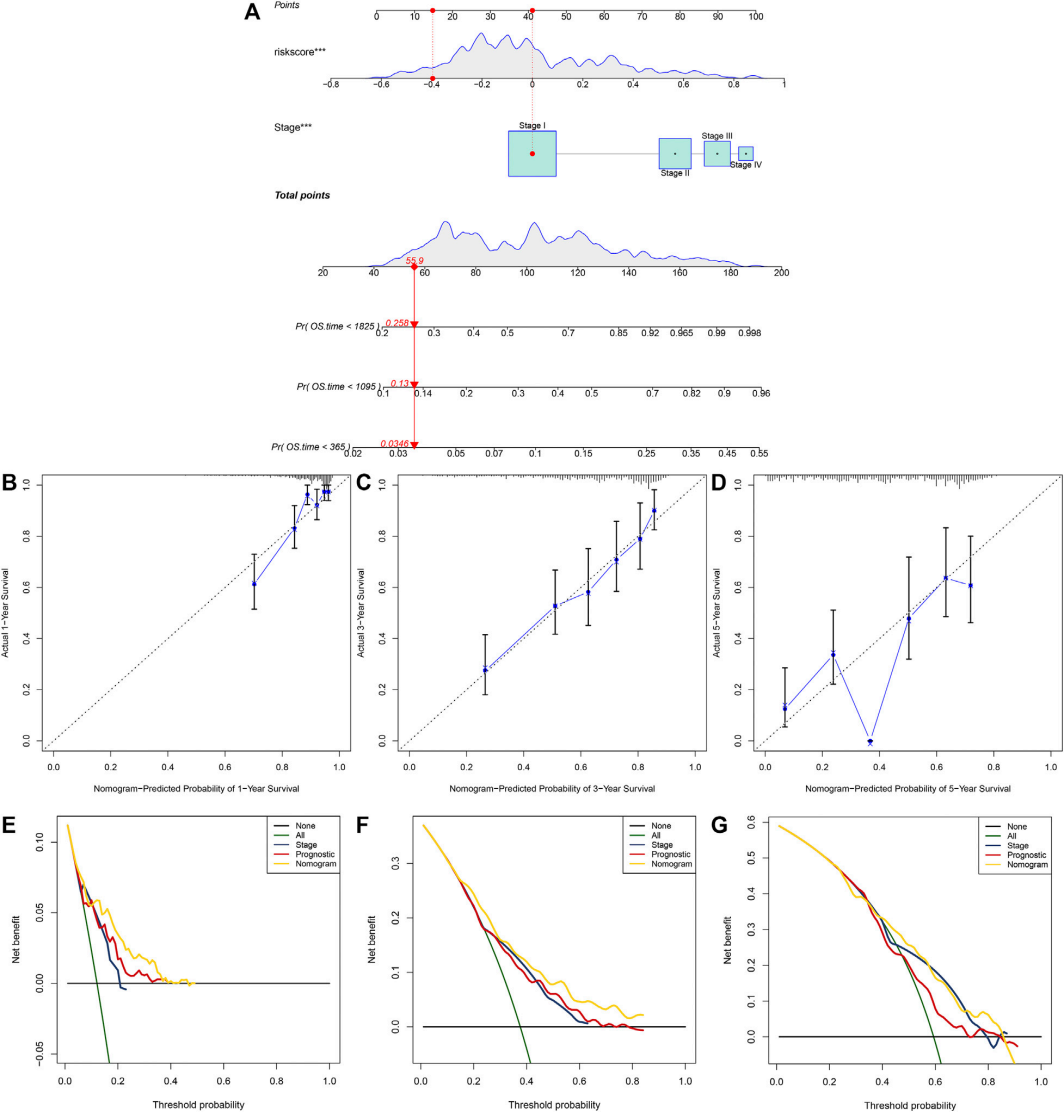
Establishment of the nomogram for predicting survival likelihood of LUAD patients in the TCGA dataset. **(A)** The nomogram that included independent prognostic indicators obtained from multivariate-cox regression analysis for predicting one, three and 5-year OS likelihood of LUAD patients. Each variable (m^6^A risk score and stage) was projected to the point scale for obtaining a value. **(B–D)** Calibration plots of the nomogram-predicted one, three and 5-year OS probabilities against the observed rates. The *x*-axis represented nomogram-predicted survival and the *y*-axis represented actual survival. **(E–G)** Decision curves for the clinical net benefit of each model in comparison to all or none strategies. The *x*-axis indicated the threshold probability, and the *y*-axis indicated the net clinical benefit.

A nomogram was built for predicting one, three and 5-year OS likelihood of LUAD patients in the TCGA dataset, including the m^6^A risk score and stage that were obtained from multivariate-cox regression analysis (Figure 8A). Calibration diagram demonstrated that there was a high consistency in this nomogram-predicted and actual one, three and 5-year OS probabilities (Figures 8B–D). Moreover, decision curves suggested that the nomogram showed the best clinical net benefit for one, three and 5-year OS (Figures 8E–G). The nomogram was externally verified in the GSE30219 cohort (Supplementary Figure S1A–G) and GSE72094 cohort (Supplementary Figure S2A–G).

## Discussion

LUAD is usually diagnosed at an advanced stage, characterized by a high mortality (Zhu et al., 2020). The development of more effective therapeutic strategies requires an in-depth understanding of factors that impact the initiation and progression of LUAD. Recently, several studies have reported the key implications of m^6^A regulators during LUAD development (Li F. et al., 2020; Chao et al., 2020; Li Y. et al., 2020). For example, Zhuang et al. constructed a diagnostic score model and a prognostic model for LUAD based on m^6^A regulators (Zhuang et al., 2020). Zhou et al. characterized two molecular subtypes with diverse prognosis and tumor microenvironment in LUAD based on m^6^A RNA methylation modification (Zhou et al., 2021). Wu et al. developed a five-m^6^A regulatory gene signature as a prognostic biomarker in LUAD patients (Wu X. et al., 2021). Xu et al. proposed m^6^A-related lncRNAs as potential biomarkers for prediction of prognosis and immune response in patients with LUAD (Xu et al., 2021). However, more studies should be presented for investigating the biological significance of m^6^A regulators in LUAD progression and prognosis. Hence, this study systematically analyzed the abnormal expression and clinical implications of m^6^A regulators.

By consensus clustering analysis, we established two m^6^A modification patterns with distinct survival outcomes based on the expression matrix of 20 m^6^A regulators for LUAD (Figure 2). Immune evasion represents a “hallmark of cancer,” which reflects that immune effector constitutes a key determinant in the tumor microenvironment (Liu J. et al., 2020). Exploring the interactions between tumors and corresponding immune cells may reveal powerful and novel treatment options against LUAD. Immunotherapies like PD1/PD-L1 inhibitors have become the standard-of-care therapeutic strategy against NSCLC (Cui et al., 2019). But, only 20–30% patients respond to such therapy (Rittmeyer et al., 2017). Limited data are available concerning the interactions between markers and immunotherapy response. Hence, it is necessary to explore and identify effective tumor immunity-related markers for LUAD, thereby reducing the mortality and developing innovative targeted therapeutic options. Compared to C2, C1 had an increased immune score and most of HLAs were highly expressed in C1, indicating that patients in C1 pattern exhibited higher tumor immunity (Figure 3).

Much progress in genome-wide methods like RNA-seq and microarrays has accelerated the evolution of cancer biomarker-related research. Numerous genetic markers of LUAD have been discovered, which are significantly correlated to diagnosis, survival outcomes, and drug resistance. But most of studies are limited to a single marker or a small sample population, leading to the limited accuracy and availability of markers. Furthermore, due to tumor heterogeneity, conventional clinical parameters like TNM staging are difficult to meet the requirement of accuracy and individuation in predicting prognosis. Thus, combined multiple markers or large sample analysis are necessary. Here, we established an m^6^A risk score containing ANLN, PLK1, IGF2BP1, HMMR, NEIL3, SFTA3, CXCL17, IRX5, PKP2, LYPD3, ABCC2, and DKK1 for LUAD prognosis (Figure 5). High m^6^A risk score indicated reduced OS duration of LUAD patients after external validation in two cohorts (Figure 6). Following multivariate-cox regression analysis, m^6^A risk score was an independent prognostic indicator of LUAD (Figure 7). ROC curves confirmed the excellent performance in predicting LUAD patients’ outcomes.

Previously, ANLN up-regulation is in relation to LUAD metastasis (Xu et al., 2019). ANLN promotes LUAD progression via activating RHOA and involving the PI3K/AKT signaling (Suzuki et al., 2005). PLK1/vimentin pathway promotes immune escape through recruitment of Smad2/3 to PD-L1 promoter in LUAD metastasis (Jang et al., 2021). PLK1 induces migration of LUAD epithelial cells through STAT3 (Yan et al., 2018). IGF2BP1 induces LUAD progression via interaction with circXPO1 (Huang et al., 2020). Up-regulation of IGF2BP1 contributes to an unfavorable prognosis of LUAD (Huang et al., 2019). HMMR acts as an oncogene of LUAD and induces tumor progression (Li W. et al., 2020). NEIL3 that is correlated to immune infiltrations serves as an independent indicator for prediction of LUAD survival (Zhao et al., 2021). CXCL17 is an important determinator for LUAD spine metastasis (Liu W. et al., 2020). IRX5 as an oncogene is in relation to LUAD outcomes (Zhang et al., 2018). PKP2 accelerates the development of LUAD through increasing focal adhesion and EMT (Wu Y. et al., 2021). Elevated expression of LYPD3 contributes to LUAD carcinogenesis and unfavorable survival outcomes (Hu et al., 2020). ABCC2, a multidrug resistance-associated protein, displays an increased expression in LUAD (Maruhashi et al., 2018). DKK1 is an immune-associated prognostic marker in LUAD (Zhang et al., 2019). Above findings revealed the critical biological implications of the variables in the m^6^A risk score in the progression of LUAD.

Furthermore, our data indicated that in comparison to the nomogram established by a single prognostic indicator, the nomogram established by the m^6^A risk score and clinical features might become the best model in prediction of short- and long-term survival of LUAD patients, thereby possibly assisting clinical management and therapy (Figure 8). However, there are some limitations in our study, as follows: firstly, more information should be provided for internal mechanisms of m^6^A modification; secondly, the prognostic value of the m^6^A risk score should be verified in prospective research.

## Conclusion

Collectively, this study comprehensively characterized the expression and clinical implication of m^6^A regulators in LUAD. Two m^6^A modification patterns were conducted, with different OS and tumor immunity. Furthermore, we developed the m^6^A risk score, which had high accuracy in predicting LUAD prognosis. Thus, our data may provide a reliable tool for prediction of prognosis and optimization of immunotherapy for LUAD patients.

## Data Availability

The original contributions presented in the study are included in the article/supplementary material, further inquiries can be directed to the corresponding author.

## Abbreviations

NSCLC, non-small cell lung cancer; LUAD, lung adenocarcinoma; m^6^A, N^6^-methyladenosine; TCGA, The Cancer Genome Atlas; GEO, Gene Expression Omnibus; GSVA, Gene set variation analysis; ESTIMATE, Estimation of Stromal and Immune Cells in Malignant Tumors Using Expression Data; HLA, human leukocyte antigen; ssGSEA, single-sample gene set enrichment analysis; DEGs, differentially expressed genes; FC, fold change; FDR, false discovery rate; LASSO, least absolute shrinkage and selection operator; OS, overall survival; ROC, receiver operating characteristic; AUC, area under curv; HR, hazard ratio.

## References

[B1] Bray F. Ferlay J. Soerjomataram I. Siegel R. L. Torre L. A. Jemal A. (2018). Global Cancer Statistics 2018: GLOBOCAN Estimates of Incidence and Mortality Worldwide for 36 Cancers in 185 Countries. CA: A Cancer J. Clinicians 68 (6), 394–424. 10.3322/caac.21492 30207593

[B2] Chao Y. Shang J. Ji W. (2020). ALKBH5-m6A-FOXM1 Signaling axis Promotes Proliferation and Invasion of Lung Adenocarcinoma Cells under Intermittent Hypoxia. Biochem. Biophysical Res. Commun. 521 (2), 499–506. 10.1016/j.bbrc.2019.10.145 31677788

[B3] Charoentong P. Finotello F. Angelova M. Mayer C. Efremova M. Rieder D. (2017). Pan-cancer Immunogenomic Analyses Reveal Genotype-Immunophenotype Relationships and Predictors of Response to Checkpoint Blockade. Cel Rep. 18 (1), 248–262. 10.1016/j.celrep.2016.12.019 28052254

[B4] Cui Y. Fang W. Li C. Tang K. Zhang J. Lei Y. (2019). Development and Validation of a Novel Signature to Predict Overall Survival in "Driver Gene-Negative" Lung Adenocarcinoma (LUAD): Results of a Multicenter Study. Clin. Cancer Res. 25 (5), 1546–1556. 10.1158/1078-0432.Ccr-18-2545 30389658

[B5] Ding Y. Qi N. Wang K. Huang Y. Liao J. Wang H. (2020). FTO Facilitates Lung Adenocarcinoma Cell Progression by Activating Cell Migration through mRNA Demethylation. Ott 13, 1461–1470. 10.2147/ott.S231914 PMC7035887 32110044

[B6] Engebretsen S. Bohlin J. (2019). Statistical Predictions with Glmnet. Clin. Epigenet 11 (1), 123. 10.1186/s13148-019-0730-1 PMC6708235 31443682

[B7] Fu Y. Dominissini D. Rechavi G. He C. (2014). Gene Expression Regulation Mediated through Reversible m6A RNA Methylation. Nat. Rev. Genet. 15 (5), 293–306. 10.1038/nrg3724 24662220

[B8] Hänzelmann S. Castelo R. Guinney J. (2013). GSVA: Gene Set Variation Analysis for Microarray and RNA-Seq Data. BMC Bioinformatics 14, 7. 10.1186/1471-2105-14-7 23323831 PMC3618321

[B9] Hu P. Huang Y. Gao Y. Yan H. Li X. Zhang J. (2020). Elevated Expression of LYPD3 Is Associated with Lung Adenocarcinoma Carcinogenesis and Poor Prognosis. DNA Cel Biol. 39 (4), 522–532. 10.1089/dna.2019.5116 32040344

[B10] Huang H. Wang D. Guo W. Zhuang X. He Y. (2019). Correlated Low IGF2BP1 and FOXM1 Expression Predicts a Good Prognosis in Lung Adenocarcinoma. Pathol. - Res. Pract. 215 (7), 152433. 10.1016/j.prp.2019.152433 31085008

[B11] Huang Q. Guo H. Wang S. Ma Y. Chen H. Li H. (2020). A Novel Circular RNA, circXPO1, Promotes Lung Adenocarcinoma Progression by Interacting with IGF2BP1. Cell Death Dis 11 (12), 1031. 10.1038/s41419-020-03237-8 33268793 PMC7710735

[B12] Jang H.-R. Shin S.-B. Kim C.-H. Won J.-Y. Xu R. Kim D.-E. (2021). PLK1/vimentin Signaling Facilitates Immune Escape by Recruiting Smad2/3 to PD-L1 Promoter in Metastatic Lung Adenocarcinoma. Cell Death Differ 28, 2745–2764. 10.1038/s41418-021-00781-4 33963314 PMC8408167

[B13] Jia Q. Wu W. Wang Y. Alexander P. B. Sun C. Gong Z. (2018). Local Mutational Diversity Drives Intratumoral Immune Heterogeneity in Non-small Cell Lung Cancer. Nat. Commun. 9 (1), 5361. 10.1038/s41467-018-07767-w 30560866 PMC6299138

[B14] Li F. Wang H. Huang H. Zhang L. Wang D. Wan Y. (2020). m6A RNA Methylation Regulators Participate in the Malignant Progression and Have Clinical Prognostic Value in Lung Adenocarcinoma. Front. Genet. 11, 994. 10.3389/fgene.2020.00994 33193582 PMC7477360

[B15] Li W. Pan T. Jiang W. Zhao H. (2020). HCG18/miR-34a-5p/HMMR axis Accelerates the Progression of Lung Adenocarcinoma. Biomed. Pharmacother. 129, 110217. 10.1016/j.biopha.2020.110217 32559619

[B16] Li Y. Gu J. Xu F. Zhu Q. Chen Y. Ge D. (2020). Molecular Characterization, Biological Function, Tumor Microenvironment Association and Clinical Significance of m6A Regulators in Lung Adenocarcinoma. Brief Bioinform 22, bbaa225. 10.1093/bib/bbaa225 33003204

[B17] Liberzon A. Birger C. Thorvaldsdóttir H. Ghandi M. Mesirov J. P. Tamayo P. (2015). The Molecular Signatures Database Hallmark Gene Set Collection. Cel Syst. 1 (6), 417–425. 10.1016/j.cels.2015.12.004 PMC4707969 26771021

[B18] Liu J. Han X. Chen L. Han D. Mu X. Hu X. (2020). TRIM28 Is a Distinct Prognostic Biomarker that Worsens the Tumor Immune Microenvironment in Lung Adenocarcinoma. aging 12 (20), 20308–20331. 10.18632/aging.103804 33091876 PMC7655206

[B19] Liu W. Xie X. Wu J. (2020). Mechanism of Lung Adenocarcinoma Spine Metastasis Induced by CXCL17. Cell Oncol. 43 (2), 311–320. 10.1007/s13402-019-00491-7 PMC12990711 31832986

[B20] Ma L. Chen T. Zhang X. Miao Y. Tian X. Yu K. (2021). The m6A Reader YTHDC2 Inhibits Lung Adenocarcinoma Tumorigenesis by Suppressing SLC7A11-dependent Antioxidant Function. Redox Biol. 38, 101801. 10.1016/j.redox.2020.101801 33232910 PMC7691619

[B21] Maruhashi R. Akizuki R. Sato T. Matsunaga T. Endo S. Yamaguchi M. (2018). Elevation of Sensitivity to Anticancer Agents of Human Lung Adenocarcinoma A549 Cells by Knockdown of Claudin-2 Expression in Monolayer and Spheroid Culture Models. Biochim. Biophys. Acta (Bba) - Mol. Cel Res. 1865 (3), 470–479. 10.1016/j.bbamcr.2017.12.005 29247669

[B22] Ritchie M. E. Phipson B. Wu D. Hu Y. Law C. W. Shi W. (2015). Limma powers Differential Expression Analyses for RNA-Sequencing and Microarray Studies. Nucleic Acids Res. 43 (7), e47. 10.1093/nar/gkv007 25605792 PMC4402510

[B23] Rittmeyer A. Barlesi F. Waterkamp D. Park K. Ciardiello F. von Pawel J. (2017). Atezolizumab versus Docetaxel in Patients with Previously Treated Non-small-cell Lung Cancer (OAK): a Phase 3, Open-Label, Multicentre Randomised Controlled Trial. The Lancet 389 (10066), 255–265. 10.1016/s0140-6736(16)32517-x PMC6886121 27979383

[B24] Rousseaux S. Debernardi A. Jacquiau B. Vitte A.-L. Vesin A. Nagy-Mignotte H. (2013). Ectopic Activation of Germline and Placental Genes Identifies Aggressive Metastasis-Prone Lung Cancers. Sci. Translational Med. 5 (186), 186ra166. 10.1126/scitranslmed.3005723 PMC4818008 23698379

[B25] Schabath M. B. Welsh E. A. Fulp W. J. Chen L. Teer J. K. Thompson Z. J. (2016). Differential Association of STK11 and TP53 with KRAS Mutation-Associated Gene Expression, Proliferation and Immune Surveillance in Lung Adenocarcinoma. Oncogene 35 (24), 3209–3216. 10.1038/onc.2015.375 26477306 PMC4837098

[B26] Schmidt L. Eskiocak B. Kohn R. Dang C. Joshi N. S. DuPage M. (2019). Enhanced Adaptive Immune Responses in Lung Adenocarcinoma through Natural Killer Cell Stimulation. Proc. Natl. Acad. Sci. USA 116 (35), 17460–17469. 10.1073/pnas.1904253116 31409707 PMC6717259

[B27] Shi Y. Fan S. Wu M. Zuo Z. Li X. Jiang L. (2019). YTHDF1 Links Hypoxia Adaptation and Non-small Cell Lung Cancer Progression. Nat. Commun. 10 (1), 4892. 10.1038/s41467-019-12801-6 31653849 PMC6814821

[B28] Suzuki C. Daigo Y. Ishikawa N. Kato T. Hayama S. Ito T. (2005). ANLN Plays a Critical Role in Human Lung Carcinogenesis through the Activation of RHOA and by Involvement in the Phosphoinositide 3-kinase/AKT Pathway. Cancer Res. 65 (24), 11314–11325. 10.1158/0008-5472.Can-05-1507 16357138

[B29] Szklarczyk D. Morris J. H. Cook H. Kuhn M. Wyder S. Simonovic M. (2017). The STRING Database in 2017: Quality-Controlled Protein-Protein Association Networks, Made Broadly Accessible. Nucleic Acids Res. 45 (D1), D362–d368. 10.1093/nar/gkw937 27924014 PMC5210637

[B30] Wilkerson M. D. Hayes D. N. (2010). ConsensusClusterPlus: a Class Discovery Tool with Confidence Assessments and Item Tracking. Bioinformatics 26 (12), 1572–1573. 10.1093/bioinformatics/btq170 20427518 PMC2881355

[B31] Wu X. Sheng H. Wang L. Xia P. Wang Y. Yu L. (2021). A Five-m6A Regulatory Gene Signature Is a Prognostic Biomarker in Lung Adenocarcinoma Patients. Aging 13 (7), 10034–10057. 10.18632/aging.202761 33795529 PMC8064222

[B32] Wu Y. Liu L. Shen X. Liu W. Ma R. (2021). Plakophilin-2 Promotes Lung Adenocarcinoma Development via Enhancing Focal Adhesion and Epithelial-Mesenchymal Transition. Cmar 13, 559–570. 10.2147/cmar.S281663 PMC7837596 33519235

[B33] Xu F. Huang X. Li Y. Chen Y. Lin L. (2021). m6A-related lncRNAs Are Potential Biomarkers for Predicting Prognoses and Immune Responses in Patients with LUAD. Mol. Ther. - Nucleic Acids 24, 780–791. 10.1016/j.omtn.2021.04.003 33996259 PMC8094594

[B34] Xu J. Zheng H. Yuan S. Zhou B. Zhao W. Pan Y. (2019). Overexpression of ANLN in Lung Adenocarcinoma Is Associated with Metastasis. Thorac. Cancer 10 (8), 1702–1709. 10.1111/1759-7714.13135 31268619 PMC6669805

[B35] Yan W. Yu H. Li W. Li F. Wang S. Yu N. (2018). Plk1 Promotes the Migration of Human Lung Adenocarcinoma Epithelial Cells via STAT3 Signaling. Oncol. Lett. 16 (5), 6801–6807. 10.3892/ol.2018.9437 30405824 PMC6202555

[B36] Yoshihara K. Shahmoradgoli M. Martínez E. Vegesna R. Kim H. Torres-Garcia W. (2013). Inferring Tumour Purity and Stromal and Immune Cell Admixture from Expression Data. Nat. Commun. 4, 2612. 10.1038/ncomms3612 24113773 PMC3826632

[B37] Zhang D.-L. Qu L.-W. Ma L. Zhou Y.-C. Wang G.-Z. Zhao X.-C. (2018). Genome-wide Identification of Transcription Factors that Are Critical to Non-small Cell Lung Cancer. Cancer Lett. 434, 132–143. 10.1016/j.canlet.2018.07.020 30031117

[B38] Zhang H. Meltzer P. Davis S. (2013). RCircos: an R Package for Circos 2D Track Plots. BMC Bioinformatics 14, 244. 10.1186/1471-2105-14-244 23937229 PMC3765848

[B39] Zhang M. Zhu K. Pu H. Wang Z. Zhao H. Zhang J. (2019). An Immune-Related Signature Predicts Survival in Patients with Lung Adenocarcinoma. Front. Oncol. 9, 1314. 10.3389/fonc.2019.01314 31921619 PMC6914845

[B40] Zhang C. Zhang G. Sun N. Zhang Z. Zhang Z. Luo Y. (2020). Comprehensive Molecular Analyses of a TNF Family-Based Signature with Regard to Prognosis, Immune Features, and Biomarkers for Immunotherapy in Lung Adenocarcinoma. EBioMedicine 59, 102959. 10.1016/j.ebiom.2020.102959 32853987 PMC7452643

[B41] Zhang H. Shi X. Huang T. Zhao X. Chen W. Gu N. (2020). Dynamic Landscape and Evolution of m6A Methylation in Human. Nucleic Acids Res. 48 (11), 6251–6264. 10.1093/nar/gkaa347 32406913 PMC7293016

[B42] Zhao C. Liu J. Zhou H. Qian X. Sun H. Chen X. (2021). NEIL3 May Act as a Potential Prognostic Biomarker for Lung Adenocarcinoma. Cancer Cel Int 21 (1), 228. 10.1186/s12935-021-01938-4 PMC8059184 33879165

[B43] Zhou H. Zheng M. Shi M. Wang J. Huang Z. Zhang H. (2021). Characteristic of Molecular Subtypes in Lung Adenocarcinoma Based on m6A RNA Methylation Modification and Immune Microenvironment. BMC Cancer 21 (1), 938. 10.1186/s12885-021-08655-1 34416861 PMC8379743

[B44] Zhu J. Wang M. Hu D. (2020). Deciphering N6-Methyladenosine-Related Genes Signature to Predict Survival in Lung Adenocarcinoma. Biomed. Res. Int. 2020, 1–13. 10.1155/2020/2514230 PMC7066421 32258108

[B45] Zhu X. Chen L. Liu L. Niu X. (2019). EMT-mediated Acquired EGFR-TKI Resistance in NSCLC: Mechanisms and Strategies. Front. Oncol. 9, 1044. 10.3389/fonc.2019.01044 31681582 PMC6798878

[B46] Zhuang Z. Chen L. Mao Y. Zheng Q. Li H. Huang Y. (2020). Diagnostic, Progressive and Prognostic Performance of m6A Methylation RNA Regulators in Lung Adenocarcinoma. Int. J. Biol. Sci. 16 (11), 1785–1797. 10.7150/ijbs.39046 32398949 PMC7211177

