# Supplementary figures and images for: Genomics and Prognosis Analysis of N6-Methyladenosine Regulators in Lung Adenocarcinoma
Source: Front Genet. 2021 Dec 9;12:746666. doi: 10.3389/fgene.2021.746666 (PMC8697852; doi:10.3389/fgene.2021.746666)

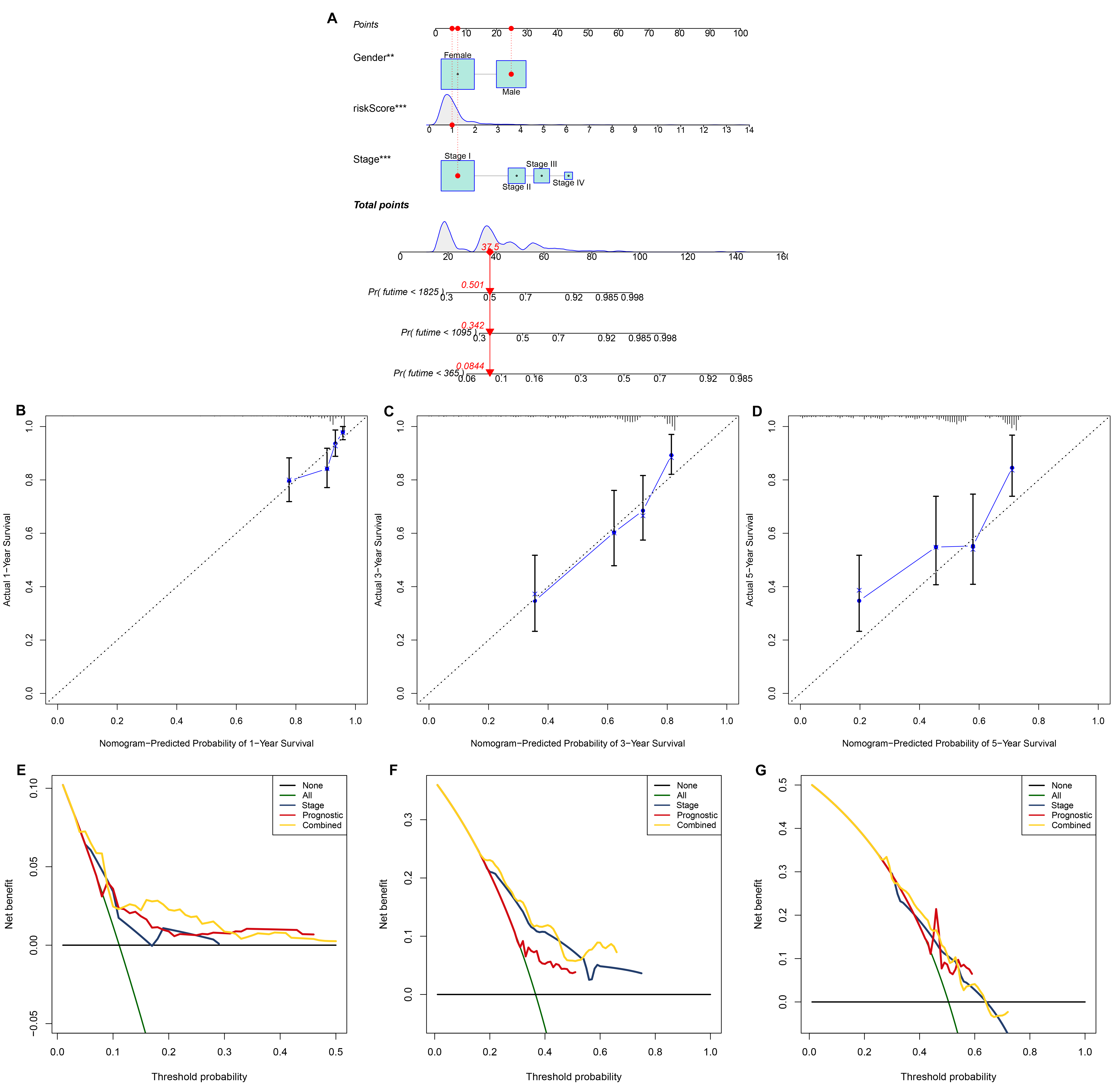

Supplement: Supplementary file 4 [file Image2.TIF]

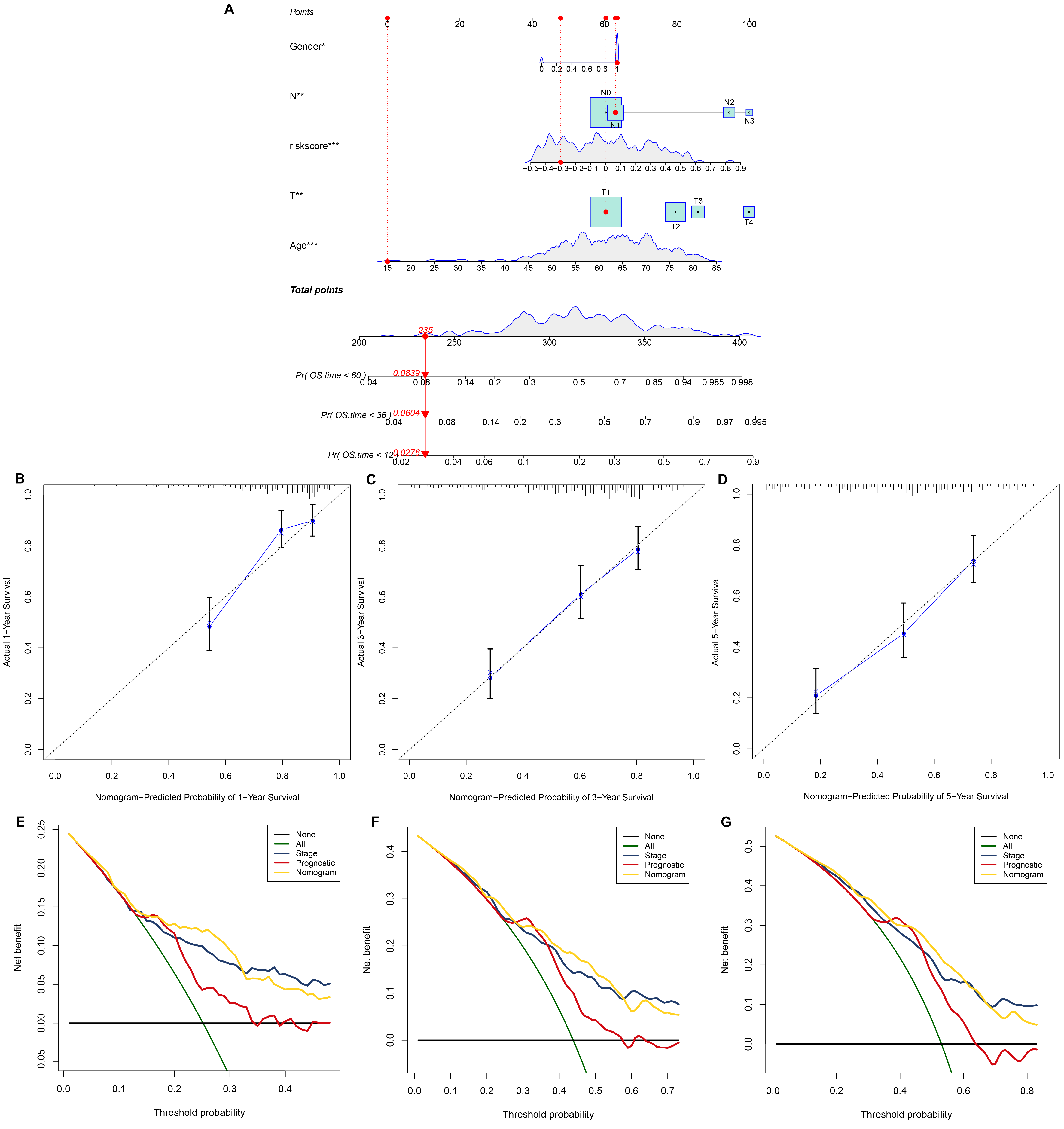

Supplement: Supplementary file 5 [file Image1.TIF]
